# The SARS-CoV-2 Exerts a Distinctive Strategy for Interacting with the ACE2 Human Receptor

**DOI:** 10.3390/v12050497

**Published:** 2020-04-30

**Authors:** Esther S. Brielle, Dina Schneidman-Duhovny, Michal Linial

**Affiliations:** 1 Department of Biological Chemistry, Institute of Life Sciences, The Hebrew University of Jerusalem, Jerusalem 91904, Israel; esther.brielle@mail.huji.ac.il; 2 The Alexander Grass Center for Bioengineering, The Hebrew University of Jerusalem, Jerusalem 91904, Israel; 3 The Rachel and Selim Benin School of Computer Science and Engineering, The Hebrew University of Jerusalem, Jerusalem 91904, Israel

**Keywords:** molecular dynamics, virus–host interactions, protein–protein complex, coronavirus evolution, ACE2, SARS-CoV-2

## Abstract

The COVID-19 disease has plagued over 200 countries with over three million cases and has resulted in over 200,000 deaths within 3 months. To gain insight into the high infection rate of the SARS-CoV-2 virus, we compare the interaction between the human ACE2 receptor and the SARS-CoV-2 spike protein with that of other pathogenic coronaviruses using molecular dynamics simulations. SARS-CoV, SARS-CoV-2, and HCoV-NL63 recognize ACE2 as the natural receptor but present a distinct binding interface to ACE2 and a different network of residue–residue contacts. SARS-CoV and SARS-CoV-2 have comparable binding affinities achieved by balancing energetics and dynamics. The SARS-CoV-2–ACE2 complex contains a higher number of contacts, a larger interface area, and decreased interface residue fluctuations relative to the SARS-CoV–ACE2 complex. These findings expose an exceptional evolutionary exploration exerted by coronaviruses toward host recognition. We postulate that the versatility of cell receptor binding strategies has immediate implications for therapeutic strategies.

## 1. Introduction

The coronavirus disease 2019 (COVID-19), initially detected in the Wuhan seafood market in the Hubei province of China [1], is caused by the SARS-CoV-2 (referred to as the COVID-19 virus for clarity). The COVID-19 virus spread within three months from its appearance to more than 200 countries, resulting in over 200,000 deaths worldwide. The COVID-19 virus is capable of human-to-human transmission and was introduced to humans in a zoonotic event [2].

Currently, seven confirmed coronavirus (CoV) species are known as human pathogens [3]. Four CoVs are endemic species in humans and cause mild respiratory symptoms, mostly in pediatric patients [4]. These are the HCoV-HKU1 and the HCoV-OC43 from the *Betacoronavirus* (BCoV) genus, and the HCoV-229E and the HCoV-NL63 from the *Alphacoronavirus* (ACoV) genus. The other human CoVs have caused severe outbreaks. The SARS-CoV (referred to as the SARS-2002 virus for clarity), is a BCoV that emerged in humans in 2002, giving rise to the severe acute respiratory syndrome (SARS) outbreak. The Middle East Respiratory Syndrome (MERS) BCoV caused an outbreak in 2012–2013. Most recently, the SARS-CoV-2, with high homology to the 2002 SARS-CoV, caused the current COVID-19 pandemic [5].

To gain access to host cells, coronaviruses rely on spike proteins, which are membrane-anchored trimers containing a receptor-binding S1 segment and a membrane-fusion S2 segment [6]. The S1 segment contains a receptor-binding domain (RBD) that recognizes and binds to a host cell receptor. The angiotensin-converting enzyme 2 (ACE2) was identified as the critical receptor for mediating the SARS-2002 entry into host cells [7,8]. The binding of the spike protein to the ACE2 receptor is a critical phase where the level of the ACE2 expressed on the cell membrane correlates with viral infectivity and governs clinical outcomes [9]. Consistent with the clinical pulmonary manifestation, ACE2 is widely expressed in almost all tissues, with the highest expression levels in the epithelium of the lung [10]. Similar to the SARS-2002 virus, the COVID-19 virus enters the host cell by its RBD binding to the host cell ACE2 receptor [11,12]. Host receptor recognition for cell entry is, however, not specified by the CoV genus classification. MERS-CoV is a member of the BCoV genus but does not recognize the ACE2 receptor [13]. In contrast, HCoV-NL63 is a member of the ACoV genus and does recognize the ACE2 receptor [14,15].

Herein, we analyze the binding of several CoV RBDs to ACE2 with molecular dynamics (MD) simulations and compare the stability, relative interaction strength, and dynamics of the interaction between the viral spike protein and the human ACE2 receptor.

## 2. Materials and Methods 

The structural model of the COVID-19 spike protein receptor-binding domain (RBD) in complex with ACE2 was generated by comparative modeling using MODELLER 9.18 [16] with the COVID-19 sequence (RefSeq: YP_009724390.1). We relied on the crystal structure of the spike protein receptor-binding domain from a SARS coronavirus-designed human strain complexed with the human receptor ACE2 (PDB 3SCI, resolution 2.9 Å) as a template for comparative modeling. The SARS-2002 spike protein RBD and the HCoV-NL63 in complex with ACE2 were taken from PDB 2AJF (resolution 2.9 Å) and 3KBH (resolution 3.3 Å), respectively. The missing residues were added in MODELLER. The MERS RBD structure was taken from the complex with the neutralizing antibody CDC2-C2 (PDB 6C6Z, resolution 2.1 Å) and structurally aligned onto the SARS-2002 RBD in complex with the ACE2 receptor. The designed SARS-CoV variant is from PDB 3SCI.

The MD simulations were performed with GROMACS 2020 software [17] using the CHARMM36m force field [18]. Each of the complexes was solvated in transferable intermolecular potential with 3 points (TIP3P) water molecules, and ions were added to equalize the total system charge. The steepest descent algorithm was used for initial energy minimization until the system converged at Fmax < 1000 kJ/(mol · nm). Then, water and ions were allowed to equilibrate around the protein in a two-step equilibration process. The first part of the equilibration was at a constant number of particles, volume, and temperature (NVT). The second part of the equilibration was at a constant number of particles, pressure, and temperature (NPT). For both of the MD equilibration parts, positional restraints of k = 1000 kJ/(mol · nm^2^) were applied to the heavy atoms of the protein, and the system was allowed to equilibrate at a reference temperature of 300 K, or reference pressure of 1 bar for 100 ps at a time step of 2 fs. Following the equilibration, the production simulation duration was 100 nanoseconds with 2 fs time intervals. Altogether 10,000 frames were saved for the analysis at intervals of 10 ps. The MD trajectories are available at ftp://ftp.cs.huji.ac.il/users/michall/covid19_trajectories/. 

We superimposed several MD snapshots on the recently solved X-ray structure (PDB 6VW1, resolution 2.7 Å [19,20,21]) of the COVID-19–ACE2 complex (Figure S1). The average root-mean-square deviation (RMSD) over the interface Cα atoms is ~1 Å. 

The interaction scores between the virus spike RBD and the ACE2 were calculated for each frame of the trajectory using the statistically optimized atomic potentials (SOAP) [22]. In the interface contact analysis, a residue–residue contact was defined based on the inter-atomic distance, with a cutoff of 4CH. The interaction buried surface area was calculated by subtracting the surface area of the protein complex from the sum of the surface areas of the individual proteins. The surface area was calculated as the solvent accessible surface [23].

## 3. Results

### 3.1. Comparable Interaction Energies for ACE2-Binding BCoVs 

The COVID-19 RBD (residues 319–529) shares a 72.8% sequence identity and high structural similarity with the SARS-2002 RBD (Table 1). In contrast, the RBD of HCoV-NL63 is only 17.1% identical to that of COVID-19, and there are no significant structural similarities between them (Figure S2). Remarkably, the RBD of MERS-CoV, which is structurally similar to that of COVID-19 (20.1% sequence identity, 65% structure similarity), recognizes a different host receptor (DPP4) for its cell entry and does not bind to ACE2 [13].

**Figure 1:**
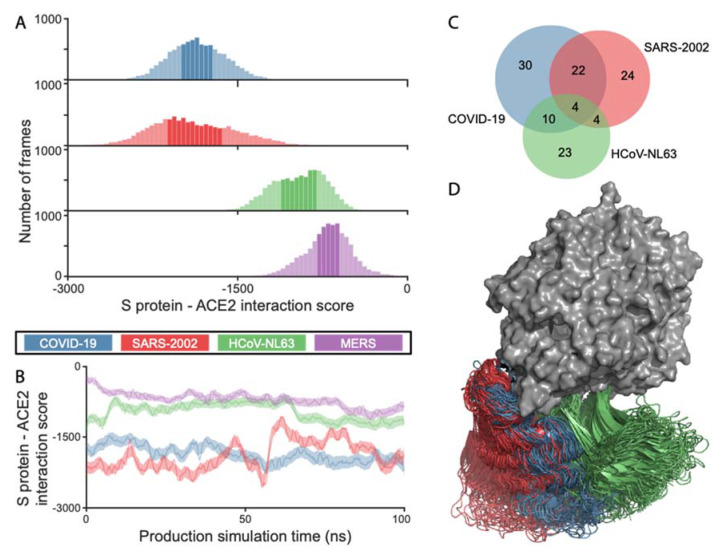
Analysis of RBD–ACE2 interactions based on the molecular dynamics (MD) trajectories. COVID-19, SARS-2002, HCoV-NL63, and MERS are colored blue, red, green, and purple, respectively. (**A**) Histograms of the RBD–ACE2 interaction scores throughout the simulation trajectory. Darker color represents 75% of all frames; (**B**) the score values along the simulation trajectory, smoothed along the elapsed time; (**C**) Venn diagram of ACE2 interacting residues for COVID-19, SARS-2002, and HCoV-NL63. An ACE2 residue is considered as part of the interface if one of its atoms is within 4 Å from any RBD atom in at least 10% of the 10,000 MD simulation frames; (**D**) overlay of 50 snapshots for each of the three RBDs. The ACE2 is in surface representation (gray). The frames were aligned using the N-terminal fragment of the ACE2 that contains the two helices participating in the RBDs binding.

We ran 100 ns molecular dynamic (MD) simulations of ACE2 in complex with the RBDs of the COVID-19, SARS-2002, and HCoV-NL63 viruses to quantify the energetics and the dynamics of the different RBD–ACE2 interactions. The simulation trajectory snapshots at 10 ps intervals (10,000 frames) were analyzed using a statistical potential to assess the probability of the RBD–ACE2 interaction (SOAP score [22]), with lower values corresponding to higher probabilities, and thus higher affinities. The interaction scores (SOAP) for COVID-19 RBD–ACE2 were comparable to those of SARS-2002, medians of −1865.9 and −1929.5, respectively (Figure 1A,B). HCoV-NL63 has RBD–ACE2 interaction scores that are higher than both of the SARS-CoVs (median of −941.6). MERS, which is structurally similar to COVID-19 (Table 1) does not bind to ACE2. The MERS virus which binds to dipeptidyl peptidase-4 (DPP4, also known as CD26 [13]), has RBD–ACE2 interaction scores that indicate an extremely weak affinity (median of −692.6), as expected from a non-cognate receptor interaction. COVID-19 has the largest buried surface area at the interface (1204 Å^2^), followed by the interface area for SARS-2002 (998 Å^2^) and HCoV-NL63 (973 Å^2^). The number of ACE2 contacting residues maintains the same order, with 30, 24, and 23 for COVID-19, SARS-2002, and HCoV-NL63, respectively (Figure 1C). The three RBDs exploit specific binding sites on ACE2 based on the analysis of the MD trajectories (Figure 1C,D, Movie S1). There is a significant overlap of ACE2 interacting residues between COVID-19 and SARS-2002 (at least 73%), while HCoV-NL63 shares only 17% and 36% of contacts with SARS-2002 and COVID-19, respectively. These findings suggest that the coronaviruses exert different interaction strategies with their cognate receptors to achieve the affinity that is required for effective cell entry.

### 3.2. Accelerated Evolution among the Key Anchoring Residues of the RBD–ACE2 Interface

**Figure 2:**
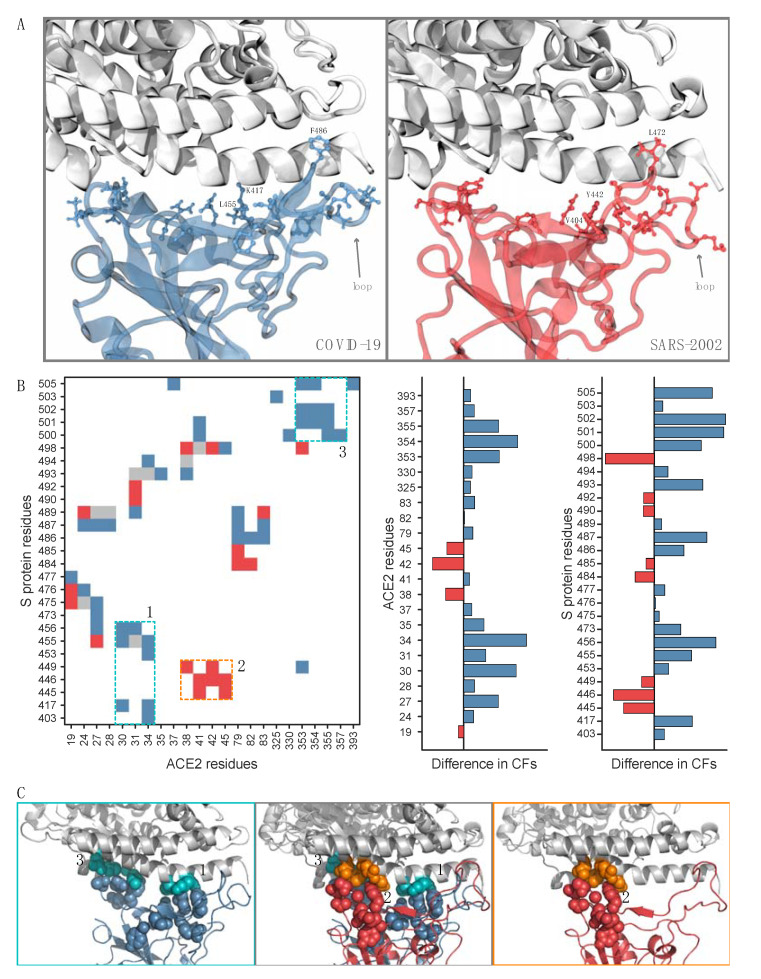
Interaction interfaces of RBD–ACE2. A residue is considered as part of the interface if one of its atoms is within 4 Å from any atom of the other partner in at least 30% of the 10,000 MD simulation frames. (**A**) The interface residue side-chain heavy atoms that vary between COVID-19 (blue) and SARS-2002 (red) are shown with ball-and-stick representations. ACE2 is colored gray; (**B**) the contacts difference plot between COVID-19 and SARS-2002 (left). A residue–residue contact frequency (CF) is defined as the fraction of MD trajectory frames in which the contact appears. The contacts with 50% greater CF in COVID-19 RBD–ACE2 vs. SARS-2002 RBD–ACE2 are colored blue. The contacts with 50% greater CF in SARS-2002 RBD–ACE2 vs. COVID-19 RBD–ACE2 are colored red. Similar interface-residue CFs (<50% difference) in both RBDs are colored gray. The residue numbering is according to COVID-19 (RefSeq: YP_009724390.1). Difference plots for interface residue CFs that are in the interface for ACE2 (middle) and RBD (right); (**C**) zooms on the interface contacts unique to each virus. The COVID-19 patches are colored blue with the corresponding ACE2 patches in cyan, and the SARS-2002 patch is in red with the corresponding ACE2 patch in orange.

While the sequence identity between the RBDs of COVID-19 and SARS-2002 is 73% (Table 1, Figure S2), we observe a significantly higher residue substitution rate at the interaction interface with the ACE2 receptor. Out of 29 RBD interface residues, only 10 residues (34%) in COVID-19 are conserved with respect to SARS-2002 (Figure 2A, Table S1, Figure S2). Similarly, only 12 residues (40%) in SARS-2002 are conserved with respect to COVID-19.

To investigate these interface residues, we constructed and overlaid the contact maps for the RBD–ACE2 interfaces for COVID-19 and SARS-2002 (Figure 2B). We define a residue–residue contact frequency (CF) as the fraction of MD trajectory frames in which the contact appears. Remarkably, only eight out of the total 72 residue–residue interface contacts have comparable (<50% difference) contact frequencies between the COVID-19–ACE2 and SARS-2002–ACE2 interfaces (Figure 2B, colored gray). Furthermore, we find two interaction patches unique to COVID-19 (Figure 2B, patches 1 and 3) and another patch unique to SARS-2002 (Figure 2B, patch 2). COVID-19 has a significant and unique contact site between the residues 500–505 of the RBD and residues 353–357 of ACE2 (Figure 2B,C). COVID-19 also creates a new interaction patch with the middle of the N-terminal ACE2 helix (Figure 2B,C), while SARS-2002 has a unique interaction patch with the end of the same helix (Figure 2B,C). The rest of the changes in the interface contact frequencies are due to the different interface loop conformations (COVID-19 residue numbers 474–498, SARS-2002 residue numbers 461–484) (Figure 2A,B, Table S1). COVID-19 has a significantly higher number of well-defined contact pairs compared to SARS-2002: 52 vs. 28 contacts (with 44 and 20 unique pairs, excluding the ones with similar CFs) were found for RBD–ACE2 of the COVID-19 and SARS-2002, respectively (Figure 2B). These results expose the accelerated evolution among the key anchoring residues of the RBD–ACE2 interface and raise the following question: how does SARS-2002 RBD reach an ACE2 binding affinity that is comparable to that of COVID-19 but with fewer contact pairs and a smaller interface area?

### 3.3. COVID-19 and SARS-2002 Differ in Their Fluctuation Signatures

The distribution of SOAP scores throughout the simulation trajectory has a larger fluctuation range for SARS-2002, relative to COVID-19 (Figure 1A,B, Figure S3A) suggesting that the SARS-2002–ACE2 interaction is fluctuating between several structural states. Moreover, the analysis of contact frequencies along the entire trajectory reveals that none of the SARS-2002 contacts are maintained over 90% of the frames, while COVID-19 still maintains about half of its contacts for 90% of the trajectory (Figure S3B).

**Figure 3:**
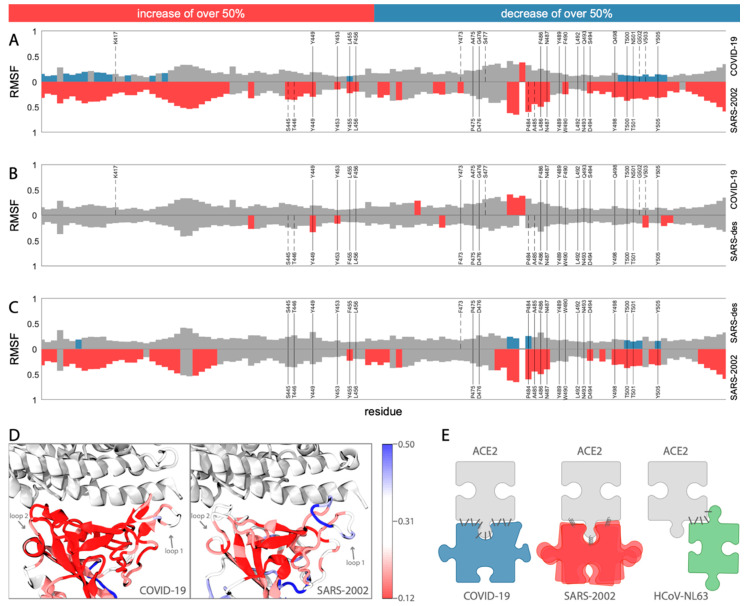
Dynamics of the RBD domains. (**A**–**C**) The dynamics of COVID-19 and SARS2002, with a comparison to a designed SARS mutant are shown. The graphs show the root-mean-square fluctuation (RMSF) of each residue along the simulation trajectory with respect to the structure with minimum energy. The residue numbers are according to COVID-19 numbering (RefSeq: YP_009724390.1). The top graph (**A**) compares COVID-19 with SARS-2002. The middle graph (**B**) compares COVID-19 with SARS-designed. The bottom graph (**C**) compares SARS-2002 with SARS-designed. For all three graphs, the positions highlighted in red or blue indicate those that have an increase or decrease, respectively, of 50% RMSF with respect to the comparison graph. The contact positions are written in gray, with solid vertical lines denoting the contact residues that exist in both of the comparison structures, and dashed vertical lines denote the contact residues that exist in only one of the comparison structures; (**D**) the RMSF mapped onto the RBD structure (red—more rigid, blue—more flexible); (**E**) the schematic representation of ACE2 binding strategies by COVID-19, SARS-2002, and HCoV-NL63 RBDs.

To investigate the dynamics of COVID-19 binding compared to SARS-2002, we calculated the root-mean-square fluctuation (RMSF) of each residue with respect to the lowest energy snapshot from their respective 100 ns MD simulation trajectory. The interface region in the RBD contains two loops (loop1: residues 474–489, loop2: residues 498–505; using COVID-19 numbering, Figure 3A,D) that bind to the ACE2 N-terminal helix on both of its ends. These two loops are highly flexible in the SARS-2002 RBD (Figure 3A,D). While loop1 is also fluctuating in the COVID-19 RBD, albeit much less, loop2 remains relatively rigid in the COVID-19 RBD. In addition, we find that in the COVID-19-RBD, a region centered around K417 leads to further stability relative to the corresponding region in SARS-2002. We attribute this difference to the unique interaction of COVID-19 at position K417 with the middle of the N-terminal ACE2 helix, thus serving as an anchor site to the receptor (Figure 2C and Figure 3A). The contribution of K417 to ACE2 binding is observed in a recent cryo-EM structure of the COVID-19 spike protein bound to ACE2 [24]. Overall, COVID-19 is more rigid compared to SARS-2002 (Figure 3A,D).

We investigated the dynamics of a designed SARS (SARS-des) variant [11], which differs from SARS-2002 at only two positions: Y455F and L486F (the corresponding positions in SARS-2002 are 442 and 472, respectively; Table S1). The L486F mutation is of special interest for the COVID-19 RBD because it has this same substitution. Our MD simulation analysis reveals that the SARS-des has a substantially lower interaction score with ACE2 (median of −2199.2, Figure S3), as expected for an optimized human ACE2-binding RBD design. We observed that these two mutations not only enhance the binding affinity to ACE2, but also lead to a substantial stabilization of the interaction interface. The fluctuation signatures along the RBD of SARS-des are surprisingly similar to those recorded for COVID-19 (Figure 3B,C). Thus, the switch from a flexible binding mode (for SARS-2002) to a stable one (COVID-19 and SARS-des, Figure 3B) highlights the remarkable capacity of the RBD to adopt alternative receptor binding strategies driven by a minimal number of amino acid substitutions. This analysis reveals the critical role of L486F (SARS-des residue F472) for stabilizing the COVID-19–ACE2 interface and producing a reduction in the number of states of the COVID-19 spike protein bound to an ACE2 receptor.

## 4. Discussion

The experimental affinity measurements (e.g., surface plasmon resonance (SPR)) confirm the high affinity of SARS-2002 RBD–ACE2 binding, with an equilibrium dissociation constant (K_D_) of ~1.5–10.0 nM [25,26,27], comparable to the binding affinity of ACE2 and the COVID-19 RBD (K_D_ = ~1.2–14.7 nM [27,28]) and consistent with our MD-based calculation (Figure 1A, Figure S3, and Table S2). While we relied on the modeled COVID-19 RBD–ACE2 interaction, the median interaction score of the complex (−1928.8), based on the MD simulation starting from the recently published X-ray structure (PDB 6LZG [21]), was even closer to the SARS-2002 RBD–ACE2 score (−1929.5). Binding affinity is achieved through a combination of interface contact optimization and protein stability (Figure 3E). While the RBD–ACE2 complex can be resolved at high-resolution by cryo-EM [24] and X-ray crystallography, MD simulations provide orthogonal information about the interaction dynamics on a nanosecond timescale. In the case of CoVs, MD simulations suggest an exceptional versatility of viral receptor binding strategies (Figure 3E). COVID-19 adopted a different strategy for achieving comparable affinity to SARS-2002: the interface of COVID-19 is significantly larger than that of SARS-2002 (1204 Å vs. 998 Å) with a remarkable number of interacting residues (ACE2: 30 vs. 24, Figure 1C). In contrast, SARS-2002 is more flexible in its interaction with ACE2, interacting through fewer contacts that serve as “hot spots”. Therefore, we predict that SARS-2002 RBD neutralizing antibodies will not be effective for COVID-19. The failure of several of these antibodies to neutralize the binding of COVID-19 RBD to its receptor is consistent with our findings [28,29]. The fluctuation from high- to low-affinity conformations in SARS-2002 led to an increased efficacy for inhibiting peptides [30] and high-affinity antibodies [31] compared to COVID-19. This implies that a therapeutic challenge is attributed to the enhanced rigidity of the COVID-19 RBD relative to that of the SARS-2002.

The geometric and physicochemical properties of RBD–ACE2 interfaces resemble those of antibody–antigen interactions. In both cases, the interface benefits from long loop plasticity, bulky aromatic side-chains as anchoring sites, and the stabilization of the complex by distributed electrostatic interactions [32]. Both COVID-19 and SARS-2002 interfaces contain long flexible loops and nine aromatic residues (Tyr, Trp, Phe) in the interface with ACE2 (Figure 2A). Moreover, in the SARS-designed variant (SARS-des [11]), the addition of an aromatic residue (L486F substitution) significantly improved the interaction scores and interface stability (Figure 3B,D). Our findings shed light on the accelerated evolution of spike protein binding to the ACE2 receptor, similar to the rapid evolution along the antibody–antigen affinity maturation process.

## Figures and Tables

**Table 1:** The sequence and structural resemblance of the receptor-binding domains (RBDs) of various human coronaviruses.

	COVID-19	SARS-2002	HCoV-NL63	MERS
**COVID-19 ^a^**	BCoV, ACE2	**72.8%**	17.1%	20.1%
**SARS-2002**	**97%** (0.9 Å)	BCoV, ACE2	17.1%	20.9%
**HCoV-NL63**	29% (4.0 Å)	29% (4.4 Å)	ACoV, ACE2	17.5%
**MERS**	**65%** (3.2 Å)	**63%** (3.3 Å)	27% (4.1 Å)	BCoV, DPP4

**^a^** The sequence identity between the RBDs is shaded blue. The structural similarity, as measured by the TM-score (*15*) between the RBD domains of the viruses, with the root-mean-square deviation (RMSD) in parentheses, is shaded red. The CoV genera and the cell entry receptors are shaded gray. The significant values are in boldface.

## Supplementary Materials

The following are available online at https://www.mdpi.com/1999-4915/12/5/497/s1, Figure S1: Comparison between the X-ray structure of COVID-19—ACE2 complex; Figure S2 Multiple sequence alignment of the analyzed RBDs; Figure S3: RBD—ACE2 interaction scores and contacts, Table S1: RBD interface residues that have substitutions between COVID-19 and SARS-2002; Table S2: Table S2. RBD—ACE2 interface evaluated by several methods for analysis of protein-protein interactions, Video S1: Overlay of 50 random snapshots from the MD trajectories of COVID-19—ACE2, SARS-2002—ACE2, and HCoV-NL63—ACE2 complexes. For clarity only one copy of ACE2 is shown (gray), COVID-19, SARS-2002, and HCoV-NL63 are colored blue, red, and green, respectively.

## References

[1] Rota P.A., Oberste M.S., Monroe S.S., Nix W.A., Campagnoli R., Icenogle J.P., Penaranda S., Bankamp B., Maher K., Chen M.H. (2003). Characterization of a novel coronavirus associated with severe acute respiratory syndrome. Science.

[2] Gralinski L.E., Menachery V.D. (2020). Return of the Coronavirus: 2019-nCoV. Viruses.

[3] Weiss S.R., Navas-Martin S. (2005). Coronavirus pathogenesis and the emerging pathogen severe acute respiratory syndrome coronavirus. Microbiol. Mol. Biol. Rev..

[4] Zeng Z.Q., Chen D.H., Tan W.P., Qiu S.Y., Xu D., Liang H.X., Chen M.X., Li X., Lin Z.S., Liu W.K. (2018). Epidemiology and clinical characteristics of human coronaviruses OC43, 229E, NL63, and HKU1: A study of hospitalized children with acute respiratory tract infection in Guangzhou, China. Eur. J. Clin. Microbiol. Infect. Dis..

[5] Wu F., Zhao S., Yu B., Chen Y.-M., Wang W., Song Z.-G., Hu Y., Tao Z.-W., Tian J.-H., Pei Y.-Y. (2020). A new coronavirus associated with human respiratory disease in China. Nature.

[6] Tortorici M.A., Walls A.C., Lang Y., Wang C., Li Z., Koerhuis D., Boons G.-J., Bosch B.-J., Rey F.A., de Groot R.J. (2019). Structural basis for human coronavirus attachment to sialic acid receptors. Nat. Struct. Mol. Biol..

[7] Kuba K., Imai Y., Rao S., Gao H., Guo F., Guan B., Huan Y., Yang P., Zhang Y., Deng W. (2005). A crucial role of angiotensin converting enzyme 2 (ACE2) in SARS coronavirus–induced lung injury. Nat. Med..

[8] Hofmann H., Geier M., Marzi A., Krumbiegel M., Peipp M., Fey G.H., Gramberg T., Pöhlmann S. (2004). Susceptibility to SARS coronavirus S protein-driven infection correlates with expression of angiotensin converting enzyme 2 and infection can be blocked by soluble receptor. Biochem. Biophys. Res. Commun..

[9] Mossel E.C., Huang C., Narayanan K., Makino S., Tesh R.B., Peters C.J. (2005). Exogenous ACE2 expression allows refractory cell lines to support severe acute respiratory syndrome coronavirus replication. J. Virol..

[10] Hamming I., Timens W., Bulthuis M., Lely A., Navis G., van Goor H. (2004). Tissue distribution of ACE2 protein, the functional receptor for SARS coronavirus. A first step in understanding SARS pathogenesis. J. Pathol. A J. Pathol. Soc. Great Br. Irel..

[11] Wan Y., Shang J., Graham R., Baric R.S., Li F. (2020). Receptor recognition by novel coronavirus from Wuhan: An analysis based on decade-long structural studies of SARS. J. Virol..

[12] Hoffmann M., Kleine-Weber H., Schroeder S., Kruger N., Herrler T., Erichsen S., Schiergens T.S., Herrler G., Wu N.H., Nitsche A. (2020). SARS-CoV-2 Cell Entry Depends on ACE2 and TMPRSS2 and Is Blocked by a Clinically Proven Protease Inhibitor. Cell.

[13] Wang N., Shi X., Jiang L., Zhang S., Wang D., Tong P., Guo D., Fu L., Cui Y., Liu X. (2013). Structure of MERS-CoV spike receptor-binding domain complexed with human receptor DPP4. Cell Res..

[14] Hofmann H., Pyrc K., van der Hoek L., Geier M., Berkhout B., Pohlmann S. (2005). Human coronavirus NL63 employs the severe acute respiratory syndrome coronavirus receptor for cellular entry. Proc. Natl. Acad. Sci. USA.

[15] Li W., Sui J., Huang I.-C., Kuhn J.H., Radoshitzky S.R., Marasco W.A., Choe H., Farzan M. (2007). The S proteins of human coronavirus NL63 and severe acute respiratory syndrome coronavirus bind overlapping regions of ACE2. Virology.

[16] Webb B., Sali A. (2014). Comparative protein structure modeling using MODELLER. Curr. Protoc. Bioinform..

[17] Pronk S., Páll S., Schulz R., Larsson P., Bjelkmar P., Apostolov R., Shirts M.R., Smith J.C., Kasson P.M., van der Spoel D. (2013). GROMACS 4.5: A high-throughput and highly parallel open source molecular simulation toolkit. Bioinformatics.

[18] Huang J., Rauscher S., Nawrocki G., Ran T., Feig M., de Groot B.L., Grubmüller H., MacKerell A.D. (2017). CHARMM36m: An improved force field for folded and intrinsically disordered proteins. Nat. Methods.

[19] Shang J., Ye G., Shi K., Wan Y., Luo C., Aihara H., Geng Q., Auerbach A., Li F. (2020). Structural basis of receptor recognition by SARS-CoV-2. Nature.

[20] Lan J., Ge J., Yu J., Shan S., Zhou H., Fan S., Zhang Q., Shi X., Wang Q., Zhang L. (2020). Structure of the SARS-CoV-2 spike receptor-binding domain bound to the ACE2 receptor. Nature.

[21] Wang Q., Zhang Y., Wu L., Niu S., Song C., Zhang Z., Lu G., Qiao C., Hu Y., Yuen K.Y. (2020). Structural and Functional Basis of SARS-CoV-2 Entry by Using Human ACE2. Cell.

[22] Dong G.Q., Fan H., Schneidman-Duhovny D., Webb B., Sali A. (2013). Optimized atomic statistical potentials: Assessment of protein interfaces and loops. Bioinformatics.

[23] Connolly M.L. (1983). Solvent-accessible surfaces of proteins and nucleic acids. Science.

[24] Yan R., Zhang Y., Li Y., Xia L., Guo Y., Zhou Q. (2020). Structural basis for the recognition of SARS-CoV-2 by full-length human ACE2. Science.

[25] Wong S.K., Li W., Moore M.J., Choe H., Farzan M. (2004). A 193-amino acid fragment of the SARS coronavirus S protein efficiently binds angiotensin-converting enzyme 2. J. Biol. Chem..

[26] Moore M.J., Dorfman T., Li W., Wong S.K., Li Y., Kuhn J.H., Coderre J., Vasilieva N., Han Z., Greenough T.C. (2004). Retroviruses pseudotyped with the severe acute respiratory syndrome coronavirus spike protein efficiently infect cells expressing angiotensin-converting enzyme 2. J. Virol..

[27] Walls A.C., Park Y.J., Tortorici M.A., Wall A., McGuire A.T., Veesler D. (2020). Structure, Function, and Antigenicity of the SARS-CoV-2 Spike Glycoprotein. Cell.

[28] Wrapp D., Wang N., Corbett K.S., Goldsmith J.A., Hsieh C.-L., Abiona O., Graham B.S., McLellan J.S. (2020). Cryo-EM structure of the 2019-nCoV spike in the prefusion conformation. Science.

[29] Tian X., Li C., Huang A., Xia S., Lu S., Shi Z., Lu L., Jiang S., Yang Z., Wu Y. (2020). Potent binding of 2019 novel coronavirus spike protein by a SARS coronavirus-specific human monoclonal antibody. Emerg. Microbes Infect..

[30] Struck A.-W., Axmann M., Pfefferle S., Drosten C., Meyer B. (2012). A hexapeptide of the receptor-binding domain of SARS corona virus spike protein blocks viral entry into host cells via the human receptor ACE2. Antivir. Res..

[31] Du L., He Y., Zhou Y., Liu S., Zheng B.J., Jiang S. (2009). The spike protein of SARS-CoV--a target for vaccine and therapeutic development. Nat. Rev. Microbiol..

[32] Kunik V., Ofran Y. (2013). The indistinguishability of epitopes from protein surface is explained by the distinct binding preferences of each of the six antigen-binding loops. Protein Eng. Des. Sel..

