# Supplementary material for: The SARS-CoV-2 Exerts a Distinctive Strategy for Interacting with the ACE2 Human Receptor
Source: Viruses. 2020 Apr 30;12(5):497. doi: 10.3390/v12050497 (PMC7291053; doi:10.3390/v12050497)
Supplement: Supplementary file 1 [file viruses-12-00497-s001.zip › viruses-779308-supplementary.pdf]

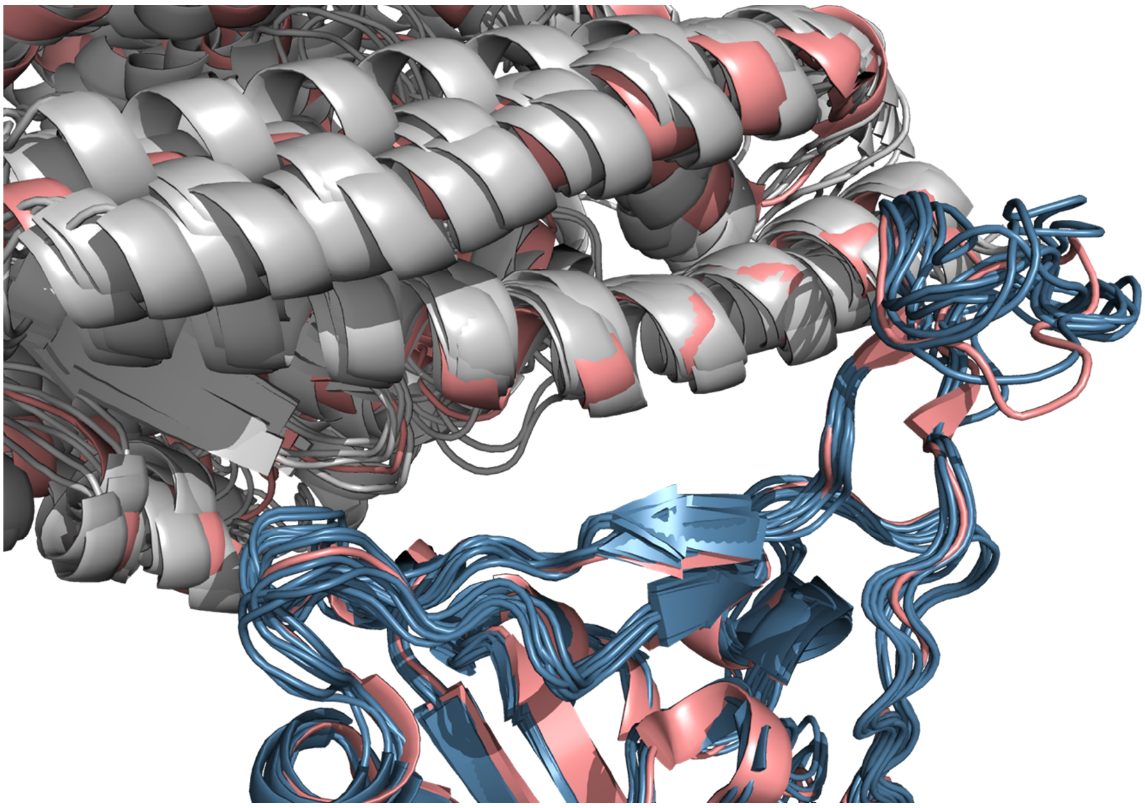

Figure S1. A comparison between the X-ray structure of the COVID-19–ACE2 complex (PDB 6VW1, pink) and the molecular dynamics (MD) trajectory frames. The MD snapshots are colored gray and blue for ACE2 and COVID-19 RBD, respectively. The average RMSD over the interface Cα atoms is ~1Å.

```
HCoV-NL63     481 QHTDINFTATASFGGSCYVCKPHQVNISLNG----------NTSVC-----------VRT
MERS          381 VECDFSPLLSGTP-PQVYNFKRLVFTNCNYNLTKLLSLFSVNDFTC-----------SYS
COVID-19      334 --CPFGEVFNATRFASVYAWNRKRISNCVADYSVLYNSASFSTFKCYGVSPTKLNDLCFT
SARS-2002     321 --CPFGEVFNATKFPSVYAWERKKISNCVADYSVLYNSTFFSTFKCYGVSATKLNDLCFS
SARS-des      321 --CPFGEVFNATKFPSVYAWERKKISNCVADYSVLYNSTFFSTFKCYGVSATKLNDLCFS
                      :.   ..:   . *  :   .. .  .          .   *             :

HCoV-NL63     520 SHFSIRYIY-----NRVKSGSPG---DSSWHIYLKSGTC---------------PFSFSK
MERS          440 SLILDYFSYPLSMKSDLSVSSAGPISQFNYKQSFSNPTCLILATVPHNLTTITKPLKYSY
COVID-19      388 NVYADSFVIRGDEVRQIAPGQTGKIADYNYKLPDDFTGCVIAWNSNNLDSKVGGNYNYLY
SARS-2002     375 NVYADSFVVKGDDVRQIAPGQTGVIADYNYKLPDDFMGCVLAWNTRNIDATSTGNYNYKY
SARS-des      375 NVYADSFVVKGDDVRQIAPGQTGVIADYNYKLPDDFMGCVLAWNTRNIDATSTGNYNYKY
                  .     :         :  ...*   : .::   .   *                 .:

HCoV-NL63     553 ----LNNFQKF----KTICFSTVE----------VPGSCNFPLEATWHYTSYTIVGALYV
MERS          500 ----INKCSRFLSDDRTEVPQLVNANQYSPCVSIVPSTV-WEDGDYYRKQLSPLEGGGWL
COVID-19      454 RLFRKSNLKPFERDISTEIYQAGS----TPCNGVEGFNCYFPLQS---YGFQPTNGVGYQ
SARS-2002     441 RYLRHGKLRPFERDISNVPFSPDG----KPCTP-PALNCYWPLND---YGFYTTTGIGYQ
SARS-des      441 RFLRHGKLRPFERDISNVPFSPDG----KPCTP-PAFNCYWPLND---YGFYTTTGIGYQ
                       .:   *     .   .                .  :           .  *  :

HCoV-NL63     599 TWSE--------------------
MERS          555 VASGSTVAMTEQLQMGFGITVQYG
COVID-19      507 PYRV--------------VVLSFE
SARS-2002     493 PYRV--------------VVLSFE
SARS-des      493 PYRV--------------VVLSFE
```

Figure S2. The multiple sequence alignment of the analyzed receptor-binding domains (RBDs).

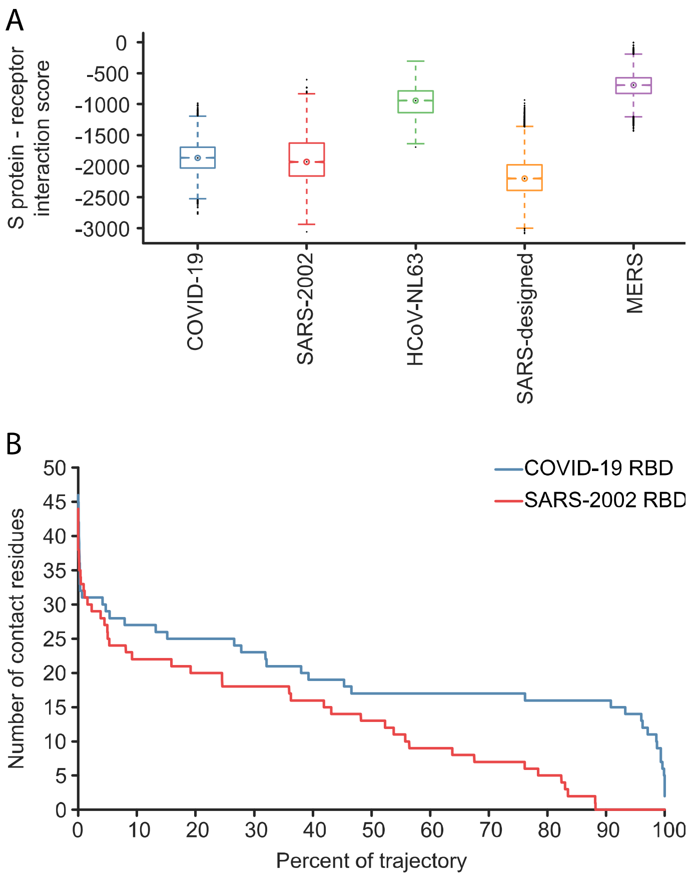

Figure S3. (A) Box plots of the statistically optimized atomic potentials (SOAP) interaction scores for the trajectory's frames. The center point is the median score, while 50% of the scores are within the box. The whiskers extend to cover > 99% of the scores. (B) The numbers of RBD residues in contact with ACE2 vs. the fraction of trajectory frames for COVID-19 (blue) and SARS-2002 (red).

COVID-19	Residue number	SARS-2002	Residue number
ARG	403	LYS	390
LYS	417	VAL	404
VAL	445	SER	432
GLY	446	THR	433
LEU	455	TYR	442
PHE	456	LEU	443
TYR	473	PHE	460
GLN	474	SER	461
ALA	475	PRO	462
GLY	476	ASP	463
SER	477	GLY	464
THR	478	LYS	465
ASN	481	THR	468
GLY	482	PRO	469
GLU	484	PRO	470
GLY	485	ALA	471
PHE	486	LEU	472
PHE	490	TRP	476
GLN	493	ASN	479
SER	494	ASP	480
GLN	498	TYR	484
PRO	499	THR	485
ASN	501	THR	487
VAL	503	ILE	489

Table S1. The receptor-binding domain (RBD) interface residues that have substitutions between COVID-19 and SARS-2002.

	PRODIGY ΔG (kcal mol^{-1})	PRODIGY Kd 10^{-9} (M)	FoldX (kcal/mol^{-1})	SOAP score
COVID-19—ACE2	-11.7	2.60	-1.93	-1865.9
SARS-2002—ACE2	-12.1	1.30	-2.67	-1929.5

Table S2. RBD–ACE2 interface evaluated by several methods for analysis of protein–protein interactions.

Movie S1. The overlay of 50 random snapshots from the MD trajectories of COVID-19–ACE2, SARS-2002–ACE2, and HCoV-NL63–ACE2 complexes. For clarity, only one copy of the ACE2 is shown (gray), and COVID-19, SARS-2002, and HCoV-NL63 are colored blue, red, and green, respectively.
